# The Indiana Medical Protection Law

**Published:** 1879-08

**Authors:** 


### THE INDIANA MEDICAL PROTECTION LAW,

Enacted in April, 1879, provides that all legally chartered medical colleges in the state shall have appointed from the State Medical Association of the same school of practice to which such college belongs, a board of examiners, who shall examine all candidates for graduation, and shall recommend or not the bestowal of diplomas. This law does not affect graduates who have been five years in practice before the passage of the act, or those not graduates who have been ten years in practice. Every other candidate for practice in the state shall present his diploma to the clerk of the court before he can obtain license to practice. The penalty for violation of the law is imprisonment in jail for not less than six months, and a fine not less than twenty-five dollars. The act is in force from its passage. This is a good law.—*Virginia Medical Monthly.*

